# Murine cancer cachexia models replicate elevated catabolic pembrolizumab clearance in humans

**DOI:** 10.1002/rco2.32

**Published:** 2021-02-09

**Authors:** Alyssa Marie M. Castillo, Trang T. Vu, Sophia G. Liva, Min Chen, Zhiliang Xie, Justin Thomas, Bryan Remaily, Yizhen Guo, Uma L. Subrayan, Travis Costa, Timothy H. Helms, Donald J. Irby, Kyeongmin Kim, Dwight H. Owen, Samuel K. Kulp, Thomas A. Mace, Mitch A. Phelps, Christopher C. Coss

**Affiliations:** ^1^ Division of Pharmaceutics and Pharmacology, College of Pharmacy The Ohio State University Columbus OH USA; ^2^ Department of Biomedical Engineering, College of Engineering The Ohio State University Columbus OH USA; ^3^ Department of Veterinary Biosciences, College of Veterinary Medicine The Ohio State University Columbus OH USA; ^4^ Division of Medical Oncology The Ohio State University James Comprehensive Cancer Center Columbus OH USA; ^5^ Division of Gastroenterology, Hepatology & Nutrition, Department of Medicine The Ohio State University Columbus OH USA; ^6^ The Comprehensive Cancer Center The Ohio State University Columbus OH 43210 USA

**Keywords:** Cachexia, Immune checkpoint inhibitor, Clearance, FcRn, C26, LLC

## Abstract

**Background:**

Monoclonal antibody (mAb) immune checkpoint inhibitor (ICI) therapies have dramatically impacted oncology this past decade. However, only about one‐third of patients respond to treatment, and biomarkers to predict responders are lacking. Recent ICI clinical pharmacology data demonstrate high baseline drug clearance (CL_0_) significantly associates with shorter overall survival, independent of ICI exposure, in patients receiving ICI mAb therapies. This suggests CL_0_ may predict outcomes from ICI therapy, and cachectic signalling may link elevated CL_0_ and poor response. Our aim was to determine if mouse models of cancer cachexia will be useful for studying these phenomena and their underlying mechanisms.

**Methods:**

We evaluated pembrolizumab CL in the C26 and Lewis lung carcinoma mouse models of cancer cachexia. A single treatment of vehicle or pembrolizumab, at a dose of 2 or 10 mg/kg, was administered intravenously by tail vein injection. Pembrolizumab was quantified by an ELISA in serial plasma samples, and FcRn gene (*Fcgrt*) expression was assessed in liver using real‐time quantitative reverse transcription PCR. Non‐compartmental and mixed‐effects pharmacokinetics analyses were performed.

**Results:**

We observed higher pembrolizumab CL_0_ and decreased *Fcgrt* expression in whole liver tissue from tumour‐bearing vs. tumour‐free mice. In multivariate analysis, presence of tumour, total murine IgG, muscle weight and *Fcgrt* expression were significant covariates on CL, and total murine IgG was a significant covariate on V1 and Q.

**Conclusions:**

These data demonstrate increases in catabolic clearance of monoclonal antibodies observed in humans can be replicated in cachectic mice, in which *Fcgrt* expression is also reduced. Notably, FcRn activity is essential for proper antigen presentation and antitumour immunity, which may permit the study of cachexia's impact on FcRn‐mediated clearance and efficacy of ICI therapies.

## Introduction

Immune checkpoint inhibitor (ICI) therapies have dramatically reshaped the therapeutic landscape across a broad range of malignancies over the past decade.^1^ To date, all seven approved ICIs are IgG monoclonal antibodies and target PD‐1 (pembrolizumab, nivolumab and cemiplimab), PD‐L1 (atezolizumab, avelumab and durvalumab), or CTLA‐4 (ipilimumab). Across the board, these drugs have radically improved the prognosis of patients with various malignancies, including non‐small cell lung cancer (NSCLC), melanoma, renal cell carcinoma, colorectal cancer, Merkel cell carcinoma, urothelial carcinoma, Hodgkin's lymphoma and primary mediastinal large B‐cell lymphoma.^2^, ^3^, ^4^, ^5^, ^6^, ^7^, ^8^, ^9^ However, despite the broad success of ICIs, only about a third of patients respond to treatment, and intensive efforts to better identify responsive patients and mechanisms of resistance are underway.^10^

In the past few years, retrospective analyses of ICI clinical pharmacology data have revealed that overall survival (OS) is shorter in NSCLC and melanoma patients with sarcopenic levels of skeletal muscle and cancer‐associated cachexia.^11^, ^12^, ^13^, ^14^, ^15^ Roch *et al*.^11^ investigated the effect of cachexia and sarcopenia on ICI efficacy in NSCLC patients receiving pembrolizumab or nivolumab as first‐line or second‐line therapy. Patients with less than 5% weight loss had improved disease control and better OS compared with their cachectic counterparts. In this same cohort, patients with evolving loss of skeletal muscle had shorter progression‐free survival and OS. Preliminary results from Shiroyama *et al*.^14^ describe similar findings in NSCLC patients, where baseline sarcopenia was significantly associated with shorter progression‐free survival. In patients with metastatic melanoma who received ipilimumab, established computed tomography methodology showed sarcopenia and low muscle attenuation were associated with more adverse events, and loss of skeletal muscle area of over 7.5%/100 days was a significant predictor of worse OS.^15^ Likewise, Chu *et al*.^12^ saw that low skeletal muscle density was prognostic of poorer outcomes of melanoma patients receiving ipilimumab and suggested that skeletal muscle density may be a better predictor of response.

Of particular interest is a retrospective analysis^16^ on the relationship between pembrolizumab pharmacokinetics (PK) and OS. Turner *et al*. revealed that among patients with advanced melanoma or NSCLC, patients with the highest pembrolizumab clearance (CL) at baseline (CL_0_) had significantly worse OS than patients with the lowest CL_0_ (8.4 vs. ~23 months median survival for the highest and lowest CL_0_ quartiles, respectively). Importantly, patients with higher CL_0_ and poorer survival developed clinical features of cancer cachexia including increased weight loss and reduced serum albumin levels. Elevated CL_0_ in these patients remained significantly associated with shorter OS even when correcting for other baseline factors associated with survival including tumour burden, performance status, tumour genetics and PD‐L1 positivity. Notably, attempts to counteract higher drug CL and consequent decreased drug exposure [both trough concentrations, C_trough_, and area under the concentration‐time curve (AUC)] with a five‐fold higher dose of pembrolizumab resulted in five‐fold higher C_trough_ and AUC, but provided no additional survival benefit to high CL patients. Together, these findings demonstrate elevated CL_0_ was strongly predictive of pembrolizumab response irrespective of circulating pembrolizumab levels, and high CL_0_ was observed more frequently in patients with cancer cachexia. In other words, cachectic patients exhibit primary resistance to ICI therapy, and the coincident higher drug CL in cachectic patients is a biomarker for, but not a cause of, this resistance.^17^ Understanding the causal mechanisms linking cachexia, higher ICI CL, and resistance to ICI therapy has become a focus in the immuno‐oncology space.^16^, ^18^, ^19^

Unlike small molecule therapeutics that are primarily metabolized and excreted, catabolism in diverse tissues throughout the body^20^ is the primary CL pathway for therapeutic IgG monoclonal antibodies.^21^ This occurs through uptake pathways, such as pinocytosis or receptor‐mediated endocytosis, in which IgG antibodies are trafficked into lysosomes and broken down into peptide fragments and individual amino acids. Both therapeutic and endogenous IgG antibodies can be salvaged from lysosomal degradation by trafficking to recycling endosomes where, under acidic conditions, they bind to the neonatal Fc receptor (FcRn), which facilitates their recycling back into the vascular space. In a similar manner, albumin homeostasis relies on FcRn for maintenance of normal serum levels.^22^ As such, FcRn‐mediated recycling is responsible for the prolonged half‐life of both IgGs and albumin, and it is well established that dysfunction or knockout of FcRn results in decreased circulating levels of both of these proteins.^22^, ^23^, ^24^ Whether cancer cachexia is associated with changes in expression of the *Fcgrt* gene, which codes for FcRn, and/or the function of FcRn protein, thereby altering IgG and albumin CL is currently unknown.

Very few reports exist describing drug PK in preclinical models of cancer cachexia, and none describe ICI monoclonal antibody PK.^25^, ^26^ In this brief report, we evaluated the utility of two commonly used models of cancer cachexia, the murine colon carcinoma (C26) and the Lewis lung carcinoma (LLC) models, in studying the clinically observed phenomena linking cachexia, ICI CL and outcomes. In particular, we focused entirely on catabolic CL, and excluded target‐mediated or antidrug antibody‐mediated monoclonal antibody CL, by evaluating single‐dose PK of PD‐1‐targeted pembrolizumab. This human IgG does not bind to murine PD‐1 but does bind to murine FcRn. While efficacy of ICI therapies has been evaluated in these models previously,^27^, ^28^, ^29^, ^30^ this is the first report to evaluate cachexia‐induced changes in ICI CL in these models and the potential mechanisms underlying these changes.

## Materials and methods

### Reagents and chemicals

Pembrolizumab (Catalogue #A2005, batch A200502) was purchased from Selleckchem (Houston, TX). *InVivoMAb* rat anti‐mouse PD‐1 (Clone RPM1‐14, Catalogue #BE0146) was purchased from BioXCell (West Lebanon, NH). Recombinant human PD‐1 Fc chimera protein and recombinant mouse PD‐1 Fc chimera protein from R&D (Minneapolis, MN) were used as capture antigens for the pembrolizumab and anti‐murine PD‐1 ELISAs, respectively. Detection antibodies conjugated to horseradish peroxidase (HRP) were purchased from BioRad (Hercules, CA), with anti‐human IgG4:HRP used for the pembrolizumab ELISA and goat anti‐rat IgG2a:HRP used for the anti‐murine PD‐1 ELISA. Wash buffer consisted of phosphate‐buffered saline (PBS) with 0.05% Tween20, and blocking solution consisted of the wash buffer solution plus 1% (w/v) bovine serum albumin, which were prepared fresh. SureBlue 3,3′,5,5′‐tetramethylbenzidine 1‐Component Microwell Peroxidase Substrate was purchased from SeraCare (Milford, MA), and 2 M H_2_SO_4_ (Millipore Sigma, Burlington, MA) was used as stop solution. TRIzol™ Reagent from ThermoFisher (Waltham, MA) was used for RNA extraction.

### Cells

Cultured murine C‐26 colon carcinoma cells and LLC cells were maintained in DMEM, supplemented with 10% foetal bovine serum and 1% penicillin–streptomycin, at 37°C in a humidified incubator with 5% CO_2_. Cell lines were tested for mycoplasma using the commercially available Plasmotest kit (Invivogen, San Diego, CA). For injection into mice, cells were harvested with trypsin, pelleted by centrifugation in foetal bovine serum‐supplemented DMEM, and then resuspended in sterile PBS at a concentration of 5 × 10^6^ C‐26 cells/mL or 10 × 10^6^ LLC cells/mL.

### Animals

Six‐week‐old to eight‐week‐old male CD2F1 mice and C57BL/6 N mice (Envigo, Indianapolis, IN) were group‐housed under conditions of constant photoperiod (12 h light/12 h dark), temperature, and humidity with *ad libitum* access to water and standard pelleted chow. Mice were weighed no less than once per week. Tumour volumes were calculated from calliper measurements using a standard formula (length × width^2^ × π/6). At the end of study, mice were euthanized by CO_2_ inhalation. All animal studies were performed in accordance with protocols approved by the Institutional Animal Care and Use Committee (IACUC) at The Ohio State University.

### Animal studies

Pembrolizumab PK parameters were determined in the C26 and LLC murine models of cancer‐associated cachexia. Three independent experiments were performed for each model with a group size of *n* = 5, except for one C26 experiment, in which group size was *n* = 3. Cachexia was established and evaluated in both models in a manner similar to that previously described.^31^ Briefly, C26 tumours were established in the right flanks of male CD2F1 mice by subcutaneous injection of 0.5 × 10^6^ C26 cells in 0.1 mL of PBS. LLC tumours were established in the right upper hind limbs of male C57BL/6 mice by intramuscular injection of 0.5 × 10^6^ LLC cells in 0.05 mL of PBS. Tumour‐free control mice received injections of sterile PBS. Twelve days after cell injection, a single treatment of PBS vehicle or pembrolizumab, at a dose of 2 or 10 mg/kg, was administered intravenously by tail vein injection. Between the two models, the total number of mice receiving 2 mg/kg of pembrolizumab was *n* = 39, and 10 mg/kg was *n* = 40. Serial blood samples were collected from the submandibular site at 1, 48 and 96 h post‐drug administration, and a terminal blood sample was taken at either 144 or 168 h after dosing by cardiac puncture immediately after euthanasia. Blood samples were collected into heparinized tubes and held at room temperature until centrifugation to collect plasma, which were stored at −80°C until analysis. Hind limb muscles (gastrocnemius, quadriceps and tibialis anterior), epididymal fat pads, tumours and liver were carefully dissected from each mouse and weighed. Sections of each tissue were placed in RNAlater (Catalogue #AM7021, ThermoFisher) and/or snap frozen in liquid nitrogen and stored at −80°C until analysis.

### Quantification of pembrolizumab in mouse plasma

Free pembrolizumab (unbound to PD‐1) was measured in mouse plasma samples by ELISA as previously described,^32^ using transparent, clear‐bottom, high binding 96‐well plates from NEST (Rahway, NJ) and the reported optimal concentrations of 0.1 μg/mL for the capture antigen and 0.2 μg/mL for the detection antibody. Washing steps were each performed three times at a volume of 300 μL/well, and all samples, standards or reagents were added at a volume of 100 μL/well. Incubation steps were performed as described with gentle shaking (300 rpm) on a microplate shaker.

Standards (ranging from 0 to 200 ng/mL) and samples were run in duplicate, and different dilution schemes were used for each dose level of pembrolizumab. For the plasma samples from mice dosed with 2 mg/kg, the 1 and 48 h samples were diluted both 1:10 000 and 1:50 000, and the 96 h and terminal samples were diluted both 1:2000 and 1:10 000. For the plasma samples from mice given 10 mg/kg, the 1 and 48 h samples were diluted 1:20 000 and 1:100 000 and the 96 h and terminal samples were diluted 1:10 000 and 1:50 000. Absorbance was measured using the Spectramax M5 plate reader (Molecular Devices, San Jose, CA) at 450 nm, corrected at 570 nm.

To ensure pembrolizumab did not bind to murine PD‐1, an ELISA plate was coated with recombinant murine PD‐1 antigen by overnight incubation at 4°C with 100 μL per well at an optimal concentration of 0.5 μg/mL. Sample processing and measurement were conducted as described above, but with the following exceptions. Pembrolizumab and anti‐murine PD‐1 were diluted in blocking buffer to create spiked calibration standards. Anti‐murine PD‐1 calibration standards at 0 to 100 ng/mL were used as positive control standards for these experiments. Detection antibody was added at a dilution of 1:5000 for anti‐murine PD‐1 standards and 1:2500 for the pembrolizumab standards at 100 μL per well for 2 h at room temperature, using the anti‐rat IgG2a‐HRP and anti‐human IgG4‐HRP antibodies, respectively.

### *Fcgrt* gene expression analyses by real‐time quantitative reverse transcription PCR

To generate liver tissue lysates, 30–60 mg of mouse liver tissue were manually ground with a pestle and lysed in 1 mL of TRIzol™ Reagent. Lysate was collected, and RNA was isolated following manufacturer's instructions for RNA extraction with TRIzol™ Reagent. High‐capacity complementary DNA reverse transcription kit (Applied Biosystems, Foster City, CA) was used to reverse transcribe total RNA (0.5 μg) to complementary DNA for 10 min at 25°C, 120 min at 37°C and 5 min at 85°C (T100™ Thermal Cycler, Bio Rad). Real‐time quantitative PCR was performed, and data were analysed and presented as previously reported, using *ACTB* (*β*‐actin) as a reference gene.^31^ Primer pairs for analyses included *FcRn*, Fd: 5′‐AGCTCAAGTTCCGATTCCTG‐3′, Rd: 5′‐GATCTGGCTGATGAATCTAGGTC‐3′, and *β‐Actin*, Fd: 5′‐AAGATCAAGATCATTGCTCCTCCTG‐3′, Rd: 5′‐AAACGCAGCTCAGTAACAGTC‐3′.

### Assessment of albumin and total IgG levels

Plasma samples collected at the terminal time point were submitted for albumin analyses to the Comparative Pathology and Digital Imaging Shared Resource at The Ohio State University.

Total plasma levels of murine IgG were detected in terminal plasma samples using an ELISA kit (Cat# 88‐50400‐22, ThermoFisher) as directed by manufacturer's protocols with the following modifications. Plasma samples were analysed in duplicate at 1:2000 and 1:10 000 dilutions for CD2F1 mice and 1:500 and 1:2000 dilutions for C57BL/6 mice. Plasma samples from a total of 73 mice receiving pembrolizumab were measured, including 35 C26 tumour‐bearing and tumour‐free control CD2F1 mice, and 38 LLC tumour‐bearing and tumour‐free control C57BL/6 mice. Statistical differences between groups were determined used Mann–Whitney tests (*P* < 0.05).

### Non‐compartmental analysis

Unbound pembrolizumab plasma concentration‐time profiles from individual mice were analysed with non‐compartmental methods using Phoenix WinNonlin (Version 8.2.0, Certara, Princeton, NJ). The terminal linear phase was chosen automatically by WinNonlin and was fit using linear least squares regression to estimate the terminal elimination rate constant (λz). AUC was determined using a linear trapezoidal linear interpolation method. Comparison of CL between tumour‐free and tumour‐bearing mice was done using Mann–Whitney test in GraphPad Prism (Version 8.0.2, GraphPad Software, San Diego, CA).

### Pharmacokinetic modelling and covariate analysis

Plasma PK data were available from 107 mice receiving human pembrolizumab. Mice erroneously receiving partial intravenous doses or having apparent/increasing absorption in the terminal phases were excluded. A total of 79 mice with PK data were available for nonlinear mixed effects analysis using NONMEM, Version 7.3, implementing the first‐order conditional estimation method with interaction (FOCE‐I).^33^ R (Version 3.3.1; http://www.r‐project.org) and the Xpose package (Version 4.5.3; http://xpose.sourceforge.net) were used for visual diagnostics.

Pembrolizumab concentration data were fit to a linear intravenous two compartment model. The model was parameterized in terms of CL, volume of distribution of the central compartment (V1), intercompartmental clearance (Q) and volume of distribution of the peripheral compartment (V2). Interindividual variability (IIV) was estimated using an exponential error model. Residual variability (ε) was described with an additive error model for log‐transformed data. Tested covariates included dose, presence of tumour, strain of mouse, terminal fat weight, terminal muscle (gastrocnemius) weight, percent body weight change, total murine IgG, albumin and *Fcgrt* (FCRN) expression. Presence of tumour, dose and strain of mouse were evaluated as dichotomous variables. Continuous covariates were normalized using population median values. Covariates having a significant influence (*P* < 0.05) were added in a forward stepwise manner until no further significant reduction in objective function was observed. Backward elimination was then performed also using a *P* value of 0.05.

## Results

### Study population

**Figure 1:**
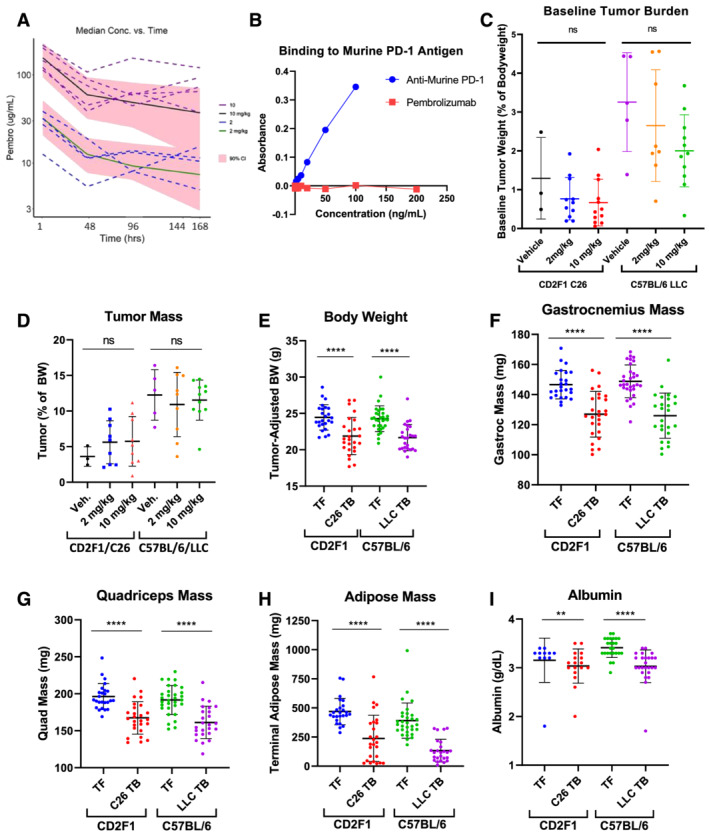
(A) Pembrolizumab pharmacokinetics. Plasma concentration vs. time profiles of pembrolizumab from all 107 mice dosed via tail vein injection at either 2 or 10 mg/kg in mice without (TF) or with tumours (TB). Pink ribbons, 95% CI of all observed data for 2 and 10/mg/kg dose groups; green and black solid lines: median of all 2 and 10 mg/kg data, respectively; blue and purple dashed lines, individual mouse curves with apparent absorption phases for 2 and 10 mg/kg dose groups, respectively. (B) Binding test of pembrolizumab to murine PD‐1. Standards of anti‐murine PD‐1 antibody (blue) from 0 to 100 ng/mL compared with pembrolizumab standards (red) from 0 to 200 ng/mL. (C) Baseline tumour volume. Baseline tumour volume (mean ± SD) as calculated from calliper measurements the day before pembrolizumab administration. Tukey's test: ns, not significant. (D–I) Characterization of tumour‐induced cachexia in the CD2F1/C26 and C57BL/6/LLC models. (D) Tumour mass (mean ± SD) as recorded after dissection from the mice, separated out into treatment groups and corrected as a percentage of the body weight (BW). (E–I) Data from all tumour‐free (TF) and tumour‐bearing (TB) mice across studies, independent of treatment group, at the time of euthanasia. (E) Tumour‐adjusted body weights, which represent the total mouse weight minus the observed or calculated tumour weights, as described in the materials and methods section. (F) Gastrocnemius mass (mean ± SD), (G) quadriceps mass (mean ± SD), (H) adipose mass (mean ± SD) and (I) albumin levels (mean ± SD). **P < 0.01; ***P < 0.001; ****P < 0.0001 by Mann–Whitney test.

A total of 135 mice were entered into these studies, including 63 C26 tumour‐bearing and tumour‐free control CD2F1 mice, and 72 LLC tumour‐bearing and tumour‐free control C57BL/6 mice. All mice were assessed for cachexia‐associated phenotypes as previously described.^31^ As pembrolizumab PK properties, and mainly CL, were our primary focus, we excluded from analyses any mice that received only a partial intravenous dose of pembrolizumab, which was suspected during dose administration and/or ultimately confirmed by obvious absorption phases between the first and second time points or assumed absorption phase in later time points in the observed drug concentration vs. time profiles (*Figure* 1A). Data from a total of 107 mice, including 25 and 26 CD2F1 mice without and with C26 tumours and 31 and 25 C57BL/6 mice without and with LLC tumours, respectively, were used for further analysis. Seventy‐nine of the 107 mice were dosed with pembrolizumab, and 28 were dosed with vehicle.

### Measurement of cachexia‐related features

The C26 and LLC mouse models of cancer cachexia are well‐established and reliably demonstrate progression of cachexia over the course of approximately 2 weeks after cell engraftment while tumours are growing. Consistent with pembrolizumab's inability to bind murine PD‐1 (*Figure* 1B), baseline tumour volumes were balanced between treatment groups in both models (*Figure* 1C), and end‐of‐study tumour weights were not significantly different within each model across treatment groups (*Figure* 1D), indicating that tumour burdens were not impacted by the single dose of pembrolizumab. Consistent with the cachectic phenotype expected in these models, tumour‐adjusted terminal body weights, skeletal muscle mass, based on gastrocnemius and quadriceps weights, adipose tissue mass and plasma albumin levels (*Figure* 1E–I) were significantly lower in tumour‐bearing than in tumour‐free mice in both models. These factors are consistent with observed cachectic phenotypes from previous cachexia studies.^31^, ^34^, ^35^

### Measurement of total murine IgG

**Figure 2:**
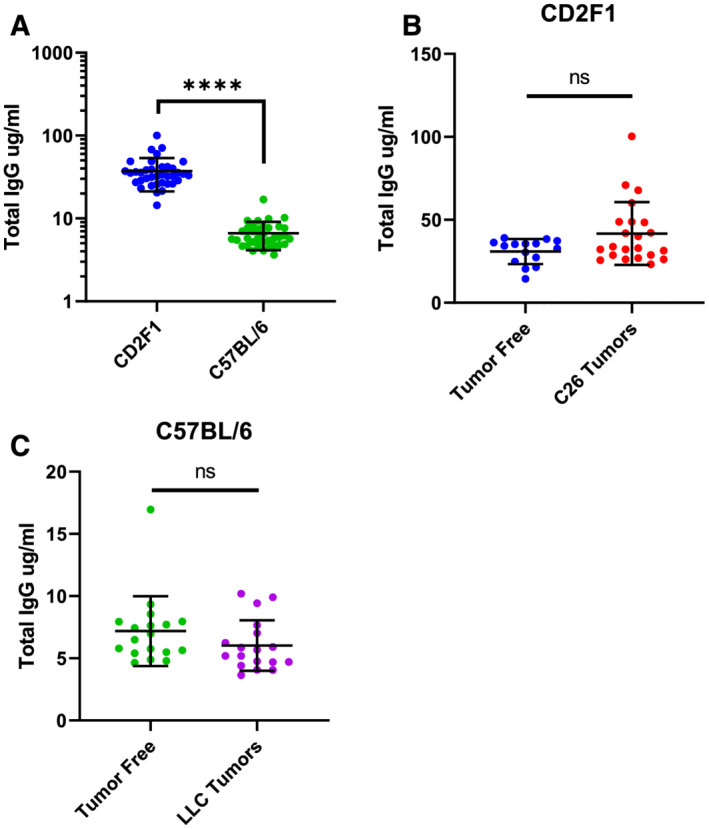
Total murine IgG levels. Total murine IgG levels measured in plasma of 73 mice receiving pembrolizumab (A) all animals, (B) CD2F1 tumour‐free or C26 tumour‐bearing, and (C) C57BL/6 tumour‐free or LLC tumour‐bearing. Mann–Whitney test: ****P < 0.001; ns, not significant.

Consistent with previous reports of similarly aged mice,^36^ total IgG levels were higher in CD2F1 (a Balb/c substrain, mean 37.328 ± 15.914 μg/mL, range 14.379–100.358 μg/mL) compared with C57BL/6 mice (mean 6.610 ± 2.451 μg/mL, range 3.647–10.187 μg/mL, *Figure* 2A). There were no statistical differences between tumour‐bearing or tumour‐free mice, though when strains were considered individually, IgG levels trended slightly higher in CD2F1 mice bearing a C26 tumour compared with tumour‐free CD2F1 mice (*Figure* 2B–C).

**Figure 3:**
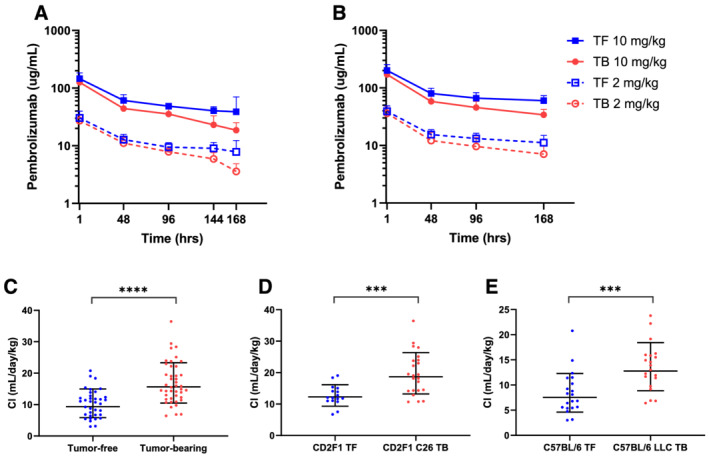
(A, B) Pembrolizumab pharmacokinetics. Data from 79 mice without apparent absorption phases. Data are combined from three independent experiments. (A) CD2F1 mice without (TF) or with C26 tumours (TB). Data are shown as mean ± SD, with *n* = 7 to 13 for all groups and time points except the last time point, where three mice were sampled. (B) C57BL/6 mice without (TF) or with LLC tumours (TB). Data are shown as mean ± SD, with *n* = 9 to 11 for all groups. (C–E). Comparison of single‐dose pembrolizumab clearance (CL) in tumour‐free and tumour‐bearing mice. (C) CL for all CD2F1/C26 and C57BL/6/LLC mice combined determined by non‐compartment analyses as described in Materials and Methods section. (D) CD2F1/C26 model CL; (E) C57BL/6/LLC model CL. Data shown as geometric mean ± geometric SD. ***P < 0.001; ****P < 0.0001 by Mann–Whitney test.

A total of 316 pembrolizumab plasma concentrations were obtained from the 79 mice receiving either 2 or 10 mg/kg of pembrolizumab. As mentioned above, mice that were identified at the time of drug administration as receiving a partial IV dose and those that exhibited an apparent absorption phase in their individual PK profiles (*Figure* 1A) were excluded from the analysis. In both cachexia models, pembrolizumab PK were similar to previous reports, though we observed differences in the concentration‐time curves between tumour‐free and tumour‐bearing mice at both the 2 and 10 mg/kg doses of pembrolizumab (*Figure* 3A–B).^37^, ^38^ Specifically, plasma pembrolizumab concentrations were lower in tumour‐bearing mice over time than in tumour‐free mice at both dose levels in both models. At 1 h after pembrolizumab administration, plasma concentrations were comparable at each dose level in tumour‐bearing and tumour‐free mice, but, over time, pembrolizumab concentrations declined more rapidly in the tumour‐bearing mice. This finding was reflected in a non‐compartmental estimation of pembrolizumab PK parameters revealing significantly higher CL in tumour‐bearing mice than in tumour‐free mice. This difference was evident when data from both C26 and LLC models were combined (15.6 ± 1.49 mL/day/kg vs. 9.36 ± 1.6 mL/day/kg in tumour‐bearing and tumour‐free, respectively, *P* < 0.0001; *Figure* 3C) or when the models were analysed separately (*Table* 1, *Figure* 3D–E).

**Table 1:** Summary of pharmacokinetic parameters of pembrolizumab in C26 & LLC mice model

Parameter	Unit	Tumour‐free		Tumour‐bearing	
		2 mg/kg	10 mg/kg	2 mg/kg	10 mg/kg
		*n* = 19	*n* = 17	*n* = 20	*n* = 23
T_1/2_	day	4.74 (1.93, 29.4)	8.15 (1.75, 19.6)	5.85 (2.07, 16.2)	5.14 (2.55, 11)
C_0_	μg/mL	33.8 (31.1)	168 (37.8)	30.7 (29.7)	143 (28.9)
Vz	mL/kg	90.1 (40)	95.3 (38.5)	121 (39.4)	124 (30.3)
Cl	mL/day/kg	9.81 (56.2)	8.87 (43.3)	14.1 (37.9)	17.1 (43)
AUC_last_	day*μg/mL	103 (26)	531 (26.6)	83.1 (24.8)	380 (30)
AUC_inf_	day*μg/mL	204 (56.2)	1130 (43.3)	142 (37.9)	583 (43)

Values are geometric mean (geometric CV%) except for T_1/2_ values are median (min, max). T_1/2_, terminal elimination half‐life; C_0_: Initial concentration; Vz, terminal volume of distribution; CL, clearance of the drug; AUC_last_, AUC from time zero to the last measurable concentration; AUC_inf_, AUC from time zero to infinity.

### *Fcgrt* expression

**Figure 4:**
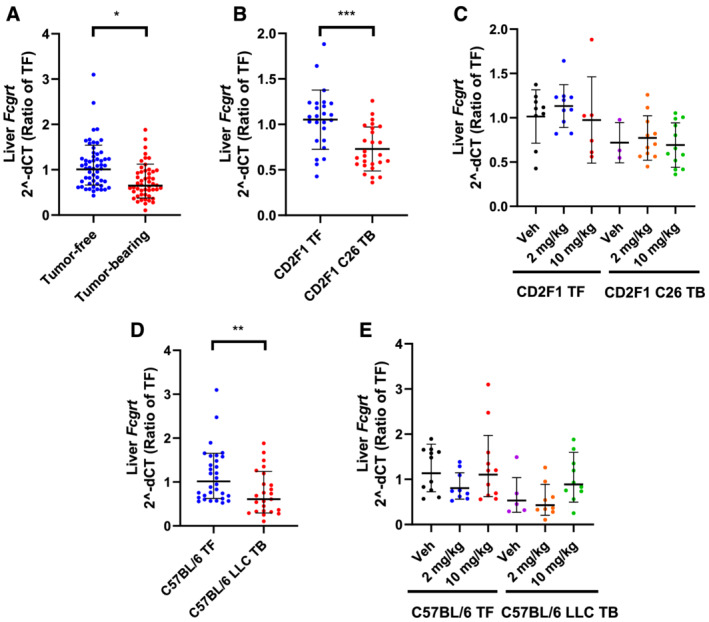
Comparison of *Fcgrt* mRNA expression in mRNA whole‐liver extracts. (A) All mice combined. (B) CD2F1/C26 TF vs. TB. (C) CD2F1/C26 separated by treatment group. (D) C57BL/6/LLC TF vs. TB. (E) C57BL/6/LLC separated by treatment group. *P < 0.05; **P < 0.01; ***P < 0.001; by Mann–Whitney test (panels A, B and D).

In light of the established role of FcRn in slowing the CL of therapeutic IgG antibodies and in the regulation of circulating albumin homeostasis, we hypothesized that elevated pembrolizumab CL and reduced albumin observed in tumour‐bearing, cachectic mice could be attributed, at least in part, to reduced FcRn expression. Liver was previously demonstrated to be a major site of IgG clearance and salvage via FcRn.^20^, ^37^ Furthermore, murine FcRn mRNA expression has been shown to be significantly correlated with FcRn protein expression.^39^ Thus, as an initial assessment of potential changes in *Fcgrt* expression in cachectic mice and the potential impact of these changes on catabolic clearance, we measured *Fcgrt* mRNA expression by real‐time quantitative reverse transcription PCR in liver homogenates from all 28 vehicle‐treated mice and 78 of 79 pembrolizumab‐treated mice in the study (one pembrolizumab‐treated animal's liver sample was lost during processing). These data demonstrate a small but significant decrease in hepatic *Fcgrt* expression in tumour‐bearing mice relative to tumour‐free animals in combined analysis of both models (*Figure* 4A). When C26 model mice were considered alone, differences between tumour‐free and tumour‐bearing mice remained (*Figure* 4B), and there was no apparent impact of treatment on *Fcgrt* expression (i.e. no statistical differences were observed within either the tumour‐free or tumour‐bearing groups, *Figure* 4C). Likewise, LLC‐tumour bearing mice had significantly reduced levels of *Fcgrt* (*Figure* 4D), and treatment with pembrolizumab had no overt effects on *Fcgrt* levels (*Figure* 4E).

### Mixed effects modelling and covariates analysis

To simultaneously evaluate the relationships between pembrolizumab PK and cachectic parameters, tumour presence, mouse strain and hepatic *Fcgrt* expression, we generated a mixed‐effects PK model. To this end, a linear intravenous two‐compartment model and a proportional error model best described the data using V1, V2, CL and Q. IIV was estimated for CL, V1 and V2 as outlined in Materials and Methods. Covariate adjusted models were developed using univariate and multivariate, stepwise selection with forward addition and backward elimination. Presence of tumour, terminal muscle weight, terminal fat weight, percent body weight change, strain of mouse, IgG and *Fcgrt* expression were significant covariates on CL, while strain of mouse, IgG, percent body weight change and *Fcgrt* expression were significant covariates on V1 in the univariate analysis (*Table* 2). Upon forward addition and backward elimination, presence of tumour, total murine IgG, terminal muscle weight and Fcgrt expression were retained as covariates on CL, and total murine IgG was retained as a covariate on V1. Notably, the categorical covariate of mouse strain was similar to the total IgG covariate with respect to its impact on the model; however, IgG was chosen for the final model given it is a continuous variable and that it may be more practical for predicting IgG monoclonal antibody clearance prospectively in other mouse strains. We also chose to retain IgG as a covariate on Q, given its significant impact on model fit (*P* < 0.0001) and despite our inability to estimate IIV on Q due to poor precision and high shrinkage of the IIV parameter. *Table* 3 summarizes the significant covariates in the final, multivariate model. The parameter estimates in the structural and final covariate model are listed in *Table* 4.

**Table 2:** Univariate analysis

Univariate covariate analysis in population model (*P* < 0.05 or *P* < 0.01)			
**Model**	**OFV**	**ΔOFV**	***P* value**
1. Base model	−559.772	—	—
2. 1 + TUM on CL	−597.974	−38.202	<0.01
3. 1 + MWT on CL	−587.179	−27.407	<0.01
4. 1 + PBWT on CL	−581.393	−21.621	<0.01
5. 1 + FWT on CL	−579.091	−19.319	<0.01
6. 1 + STR on V1	−575.994	−16.222	<0.01
7. 1 + IGG on CL	−575.258	−15.486	<0.01
8. 1 + STR on CL	−574.19	−14.418	<0.01
9. 1 + IGG on V1	−572.492	−12.72	<0.01
10. 1 + IGG on Q	−568.337	−8.565	<0.01
11. 1 + FCRNTFM on CL	−567.684	−7.912	<0.01
12. 1 + FCRN on V1	−567.32	−7.548	<0.01
13. 1 + PBWT on V1	−564.159	−4.387	<0.05

CL, clearance; FCRN, liver FCRN 2^‐ΔCT; FCRNTFM, liver FCRN expression ratio of tumour free mice; FWT, terminal fat mass; IGG, total murine IgG; MWT, terminal gastrocnemius weight; PBWT, percent body weight change; STR, strain of mouse (CD2F1 or C57BL/6); TUM, presence of tumour, yes or no; OFV, objective function value; ΔOFV, difference of objective function value.

**Table 3:** Covariate analysis with forward addition and backward elimination.

Forward addition (*P* < 0.05 or *P* < 0.01)			
**Model**	**OFV**	**ΔOFV**	***P* value**
1. Base model	−559.772	—	—
2. 1 + TUM on CL	−597.974	−38.202	<0.01
3. 2 + MWT on CL	−604.438	−6.464	<0.05
4. 3 + IGG on CL	−622.139	−17.701	<0.01
5. 4 + IGG on V1	−634.726	−12.587	<0.01
6. 5 + IGG on Q	−647.361	−12.635	<0.01
7. 6 + FCRNTFM on CL	−651.310	−3.949	<0.05

CL, clearance; FCRNTFM, liver FCRN expression ratio of tumour free mice; IGG, total murine IgG; MWT, terminal gastrocnemius weight; TUM, presence of tumour, yes or no; OFV, objective function value; ΔOFV, difference of objective function value.

**Table 4:** Population parameter estimates from the structural and final covariate model

**Parameters**	Structural model		Covariate model			
	**Estimate**	**SE, %**	**IIV, CV%**	**Estimate**	**SE, %**	**IIV, CV%**
CL (mL/day/kg)	11.9	8.5	55.0	8.35	12.3	29.6
V1 (mL/kg)	61.8	3.6	30.2	61.6	3.6	27.6
Q (mL/day/kg)	62.3	17.8	—	61.1	14.7	—
V2 (mL/kg)	62.3	6.8	50.7	66.1	6.9	51.9
ε (proportional)	0.110	8.8	—	0.110	8.6	—
**Covariates**						
TUM on CL	—	—	—	0.65	34.2	—
MWT on CL	—	—	—	−1.04	30.9	—
IGG on CL	—	—	—	0.286	18.4	—
IGG on V1	—	—	—	0.116	31.4	—
IGG on Q	—	—	—	0.415	34.9	—
FCRNTFM on CL	—	—	—	−0.227	52.4	—

CL, clearance; CV%, coefficient of variation; FCRNTFM, liver FCRN expression ratio of tumour free mice; IGG, total murine IgG; IIV, interindividual variability; MWT, terminal gastrocnemius weight; Q, intercompartmental clearance; SE, standard error; TUM, presence of tumour, yes or no; V1, volume of central compartment; V2, volume of peripheral compartment.

For ε, estimates are represented as standard deviations.

## Discussion

In this brief report, we evaluated the utility of murine models of cancer‐associated cachexia in studying underlying mechanisms of the well‐known clinical phenomenon of increased antibody drug CL in patients with cachexia. In particular, this phenomenon is observed as a significant prognostic factor for outcomes of ICI therapy in several types of cancer. While increased clearance of therapeutic antibodies in patients with cachexia and muscle wasting disease has been recognized for decades, the underlying mechanisms driving elevated therapeutic antibody CL have not been studied and remain unknown. We therefore explored changes in antibody drug CL in two well‐characterized mouse models of cancer cachexia, the C26 and LLC models.

In this study, we specifically focused on catabolic drug CL and avoided target‐mediated CL by using pembrolizumab, which is an engineered human IgG monoclonal antibody that binds specifically to human PD‐1, interacts with murine FcRn, and does not bind murine PD‐1, as we and others have confirmed.^38^ Our data demonstrated that in both animal models, tumour‐bearing, cachectic mice had a significantly higher CL of pembrolizumab compared with tumour‐free mice without cachexia. Furthermore, it is well established that when circulating levels of therapeutic monoclonal antibody far exceed high affinity drug target levels, as is the case in our studies, catabolic CL becomes the predominant means of monoclonal antibody CL.^40^ As further support that target‐mediated CL is negligible in our studies, dose level was not a significant covariate in our mixed‐effects analysis (*Table* 3). Another known contributor to monoclonal antibody CL is the formation of antidrug antibodies,^41^ though they are most common following repeat dosing and require extended time to circulate at high levels. We did not explicitly measure antidrug antibodies in our studies but given we performed short, single‐dose studies, we deemed their contribution to the observed changes in pembrolizumab CL as insignificant. When considered together, our study design features support the isolation of catabolic CL among other potential clearance pathways and, to our knowledge, this is the first study to report changes in catabolic CL of IgG monoclonal antibodies in tumour‐bearing mice with cachexia.

Given that the increased CL is observable with a single dose of pembrolizumab, we sought to determine other factors that may be associated with the increased CL. Notably, this phenomenon was not dependent on dose level, as we observed the same tumour/cachexia‐associated effect on CL at both 2 and 10 mg/kg dose levels. We also did not observe significant correlations between clearance and tumour size. However, several factors were associated with CL in univariate mixed effects modelling, including presence of tumour, mouse strain, total murine IgG, body weight, muscle mass, fat mass and liver *Fcgrt* expression. In multivariate analysis, presence of tumour, total murine IgG, terminal muscle weight and liver *Fcgrt* expression were significant covariates on CL, and total murine IgG was also significant on V1 and Q. Notably, mouse strain and total murine IgG were very similar in terms of their impact on the model, though we ultimately retained IgG as a continuous covariate and potentially more useful for prospectively predicting antibody drug PK in other mouse strains. Depending on the order of forward addition and backward elimination, either terminal muscle weight or terminal fat weight was significant on CL as well.

Our main goal in conducting these studies was to determine the relevance and potential utility of murine cachexia models for exploring underlying mechanisms associating cachexia and elevated CL of therapeutic monoclonal antibodies. These data demonstrate that at least in these two common murine models of cancer cachexia, C26 and LLC, catabolic CL of a single dose of a humanized antibody is in fact elevated in tumour‐bearing cachectic mice, apparently replicating the phenomenon observed in cachectic human cancer patients. Given the important role of FcRn in salvaging IgG from lysosomal degradation, a secondary goal was to evaluate the possibility that FcRn expression is altered in the cachectic state in these mice. In an initial assessment of this hypothesis, we evaluated *Fcgrt* gene expression in liver, which is a major catabolic clearance organ for antibodies.^20^, ^42^ Our data demonstrate a small but significant downregulation of *Fcgrt* in both the C26 and LLC tumour‐bearing mice compared with their respective CD2F1 and C57BL/6 tumour‐free control groups. Though we have thus far only assessed *Fcgrt* mRNA, this downregulation could explain at least a portion of the observed increase in pembrolizumab CL, as decreased FcRn expression is known to reduce endosomal recycling and increase antibody degradation in lysosomes. Liver *Fcgrt* expression was also retained in multivariate analysis, indicating its independence relative to other retained covariates. Notably, our work to date does not address *Fcgrt* expression in other tissues of potential significance for catabolic CL, such as skeletal muscle and spleen^43^; these and other tissues should be further explored for *Fcgrt* expression. Moreover, evaluation of changes in FcRn abundance at the protein level and assessments of FcRn function in the context of cachexia are important directions for further investigation and critical to substantiate the potential impact of the changes in mRNA levels we report. Additionally, because FcRn exists as a heterodimer with beta(2)‐microglobulin (B2M), and it has been shown that B2M plays a vital role in FcRn function and the clearance of IgGs,^44^, ^45^ further investigation into B2M expression and function will need to be performed. It is worth noting that we also observed decreased serum albumin in cachectic mice, which is consistent with decreased FcRn expression and supports the presence of a potential functional consequence of reduced *Fcgrt* expression.

In addition to *Fcgrt* expression status, several other variables were associated with altered PK and increased antibody CL in univariate and multivariate analyses. The two most prominent factors were presence of tumour and either mouse strain or total murine IgG. Use of the C26 and LLC models does not enable separation of the effects of tumour presence and cachexia (i.e. cachexia is driven by the presence of tumour in these models). However, terminal muscle weight (or terminal fat weight, which had a similar impact on the model) was also found to be a significant covariate on CL in multivariate analysis, suggesting the cachectic phenotype impacts CL independent of strain or tumour. Furthermore, while our analysis indicates presence of tumour, as a categorical variable (tumour vs. no tumour) is a highly significant factor in covariate analysis (ranked highest in terms of model impact in univariate analysis), tumour size as a continuous variable did not explain any additional variability in clearance or volume parameters within the model. Nonetheless, further investigation is needed to determine if tumour burden in the absence of cachexia will also result in increased catabolic CL of antibodies in other immune competent mouse tumour models.

In addition to tumour presence, our observations indicated that mouse strain or total murine IgG concentrations in plasma significantly impacted CL as well as V1. Given the dramatic differences in total IgG between the two strains, it is not possible to separate out these two variables in our analysis. Although previous studies have demonstrated differences among mouse strains with respect to baseline IgG levels^36^ and immune response,^46^ the apparent significance of mouse strain or total endogenous IgG on antibody disposition was unexpected. It is tempting to interpret these findings as evidence of an interaction between endogenous IgG and pembrolizumab, perhaps for binding to and salvage by FcRn. However, we generally expect FcRn capacity to be much higher than circulating IgG levels, and if there were competition between endogenous IgG and pembrolizumab for FcRn binding, one might expect to see a difference in CL between the two dose levels of pembrolizumab, which was not observed in our study. Furthermore, as FcRn salvages IgG, and if decreased Fcgrt and FcRn expression are contributing to increased pembrolizumab CL in these models, we may also expect to see decreased IgG in tumour‐bearing vs. tumour‐free mice. However, as has been previously demonstrated, IgG production may be increased in mice due to an immune response to the tumour.^47^ Without knowing how endogenous IgG production rates may differ between the two strains and how the presence of the C26 tumour in CD2F1 mice may impact endogenous IgG production rates compared to LLC tumours in C57BL/6 mice, we are not able to speculate further about the potential role of IgG levels in pembrolizumab clearance within our studies. Collectively, these findings raise additional questions regarding endogenous IgG that will need to be probed in future studies. Overall, our data support the use of the C26 and LLC mouse models for further study of the underlying mechanisms influencing increased catabolic CL of therapeutic antibodies in cancer‐associated cachexia.

As we and others pursue a better understanding of the causes for increased therapeutic antibody CL in cachectic patients, the C26 and LLC models will likely be useful. Further investigation should also include the utility of these and other models of cachexia for understanding the apparent link between cachexia and resistance to ICI therapies.^16^, ^17^, ^48^ It is tempting to speculate that cachexia‐dependent changes in FcRn underlie changes in both ICI disposition and ICI response given the established role of dysfunctional antigen presentation in primary resistance to ICIs^10^ and the evidence that normal FcRn function is critical for proper antigen presentation^49^ and antitumour immunity.^50^ Our future studies will focus on the relationship between FcRn, ICI disposition and ICI response in these now validated pre‐clinical models and expand to characterizing this interaction in cancer patients receiving ICI therapy.

## Conflict of interest

The Ohio State University James Cancer Hospital and Solove Research Institute receives research funding from Merck, Bristol Myers Squib, Palobiofarma, Genentech and Abbvie. DHO serves on a Consultant/Advisory Board for AstraZeneca. All other authors have no conflicts of interest to report.
